# Neurocognitive, Emotional, and Behavioral Costs for Adolescents Due to Diminished Returns of Parental Employment on Trauma

**DOI:** 10.31586/ojn.2025.1263

**Published:** 2025-01-24

**Authors:** Shervin Assari, Hossein Zare

**Affiliations:** 1 Department of Internal Medicine, Charles R. Drew University of Medicine and Science, Los Angeles, CA, United States; 2 Department of Family Medicine, Charles R. Drew University of Medicine and Science, Los Angeles, CA, United States; 3 Department of Urban Public Health, Charles R. Drew University of Medicine and Science, Los Angeles, CA, United States; 4 Marginalization-Related Diminished Returns (MDRs) Center, Los Angeles, CA, United States; 5 Department of Health Policy and Management, Johns Hopkins Bloomberg School of Public Health, Baltimore, MD, United States; 6 School of Business, University of Maryland Global Campus (UMGC), Adelphi, MD, United States

**Keywords:** Ethnic Groups, Disparities, Social Determinants, Child Development, Tobacco Use

## Abstract

**Background:**

Parental employment is a significant social determinant of children’s developmental outcomes, shaping their cognitive and behavioral trajectories. However, the effects of parental employment may not be equally protective across racial groups. The Minority Diminished Returns (MDRs) framework suggests that socioeconomic status (SES) factors, such as employment, yield fewer protective benefits for Black families compared to White families.

**Objective:**

This study investigates the diminished returns of parental employment on trauma and associated neurocognitive and behavioral outcomes in children, with a focus on racial variation in these effects.

**Methods:**

Using data from the Adolescent Brain Cognitive Development (ABCD) study, a large and diverse sample of children was analyzed. We applied MDRs theory and social determinants of health frameworks to examine the association between parental employment, trauma, and children’s cognitive and behavioral outcomes. The analysis controlled for family SES, neighborhood factors, and racial group differences.

**Results:**

Preliminary findings suggest that while parental employment is generally protective against trauma, the strength of this association is diminished for Black children. Black families with employed parents experience higher levels of trauma and stress compared to their White counterparts, which may contribute to racial disparities in cognitive and behavioral outcomes.

**Conclusion:**

Parental employment may not equally buffer against trauma-related risks for Black children, reflecting the broader pattern of diminished returns for racially disadvantaged groups. These findings highlight the need for policies addressing the unequal benefits of SES across racial groups.

## 1. Introduction

Parental employment is a major social determinant of children’s development, significantly influencing their cognitive, emotional, and behavioral outcomes [1–5]. Stable employment provides financial resources, social support, and psychological stability, which can protect children from a range of risks, including poverty and associated negative outcomes. Children of unemployed parents, in contrast, are at a heightened risk of experiencing poverty, which can negatively impact their development across multiple domains. This dynamic may highlight the critical role of employment in shaping the early life environment and long-term opportunities for children [5].

The broader framework of social determinants of health underscores the significance of SES indicators, such as income and employment, as fundamental causes of health and developmental outcomes. According to these theories, higher SES generally offers protection to families by providing access to better resources, healthcare, and environments that promote healthy development. However, not all families benefit equally from these SES resources. The Minority Diminished Returns (MDRs) framework [6,7] suggests that for families racialized as Black, the protective effects of SES, such as employment, are often weaker than for those racialized as White. This means that despite similar levels of SES, Black families may experience less benefit, reflecting systemic inequalities and the additional burdens of racism and discrimination.

Much of the existing research on MDRs has focused on the role of education, particularly the effects of parental education on child outcomes. However, less is known about the potential diminished returns of parental employment. Understanding how employment impacts families differently based on race is critical for addressing persistent disparities in health and development.

One possible mechanism for these diminished returns is trauma. Trauma, particularly stemming from stress and discrimination, may be more prevalent in high-SES Black families compared to their White counterparts. Despite financial and social resources, Black families often face higher levels of chronic stress due to structural racism, which may mitigate the protective effects of employment. As a result, children in these families may experience higher rates of trauma, which in turn affects their cognitive and behavioral development.

In this study, we build on MDRs theory [8] and social determinants of health frameworks [9–14] to examine how parental employment influences trauma and related cognitive and behavioral outcomes in children, focusing on racial disparities. Leveraging the diverse sample of the Adolescent Brain Cognitive Development (ABCD) study [15–23], we aim to test the hypothesis that the protective effects of parental employment on trauma are diminished for Black children compared to White children. By understanding these dynamics, we seek to contribute to the development of policies and interventions that can address racial inequalities in children’s developmental trajectories.

## 2. Methods

### 2.1. Settings and Design

This study utilized data from the Adolescent Brain Cognitive Development (ABCD) Study [15–23], a large-scale, longitudinal research initiative designed to examine brain development and child health in the United States. The ABCD study recruited over 11,000 children aged 9–10 years from 21 sites across the country, using a multi-stage probability sampling method to ensure a diverse and representative sample. Data collection involved a combination of neuroimaging, behavioral assessments, and questionnaires completed by both children and their parents. The current analysis leverages cross-sectional baseline data from the ABCD study [15–23], focusing on brain structure, socioeconomic factors, and demographic variables.

### 2.2. Sample and Sampling

The initial sample for the ABCD study [15–23] included 11,878 children. For this analysis, we restricted the sample to children identified by their parents as either Black or White, consistent with our focus on racial disparities. To ensure reliable estimates of brain structure, children with missing or poor-quality neuroimaging data were excluded from the sample.

### 2.3. Eligibility for the Current Analysis

Eligibility for inclusion in the current analysis required that children meet the following criteria: (1) aged 9–10 years at baseline, (2) identified as Black or White, (3) completed parental questionnaires on socioeconomic factors, and (4) had complete neuroimaging data for total cortical volume. Children with major neurological or psychiatric conditions or those with missing key demographic or socioeconomic data were excluded from the analysis.

### 2.4. Measures

#### Outcome (Total Cortical Volume):

The primary outcome of interest was total cortical volume, measured using MRI data collected as part of the ABCD study’s neuroimaging protocol. Cortical volume was calculated by summing the volumes of cortical gray matter across both hemispheres, using FreeSurfer software for brain image processing.

#### Predictor (Parental Education):

The key independent variable was parental education, measured as the highest level of education completed by either parent. This was treated as a continuous variable, representing years of completed education.

#### Mediator (Financial Strain):

Financial strain was measured using a parental questionnaire that assessed the family’s ability to meet basic financial needs. Questions included whether the family had difficulty paying bills, affording food, or covering medical expenses. Responses were coded to create a continuous financial strain score, with higher values indicating greater financial difficulties.

### 2.5. Covariates

We controlled for several demographic variables that could influence brain development, including the child’s age, sex, household income, and parental marital status. Household income was treated as a continuous variable, while marital status was categorized as married or not married. Age and sex were included to account for normal developmental differences in cortical volume across children.

### 2.6. Statistical Analysis

We employed Structural Equation Modeling (SEM) to test the associations between parental employment and various emotional, behavioral, and cognitive outcomes, with trauma as a potential mediator and race as a moderator. Three models were specified to evaluate the direct and indirect effects of parental employment: All analyses were conducted using SEM in Stata, and we used maximum likelihood estimation to account for missing data. Model fit was assessed using standard indices such as the Comparative Fit Index (CFI) and Root Mean Square Error of Approximation (RMSEA). Significance was evaluated at p < .05.

### 2.7. Ethics

The ABCD study received approval from the Institutional Review Boards (IRBs) at each of the 21 data collection sites. Informed consent was obtained from all parents or legal guardians, and assent was obtained from children before participation. This study’s secondary analysis of de-identified ABCD data was exempt from an full IRB review by Charles R Drew University of Medicine and Science.

## 3. Results

Table 1 provides the descriptive statistics for the key study variables. The average age of the children in the sample was 9.48 years (SE = 0.005), with a 95% confidence interval ranging from 9.47 to 9.49. In terms of race, 72.9% of the sample identified as White (SE = 0.006), and 27.1% identified as Black (SE = 0.006). The sample was fairly balanced by gender, with 47.6% of participants identifying as female (SE = 0.007) and 52.4% as male (SE = 0.007). Regarding the marital status of the household, 34.0% of children lived in an unwed household (SE = 0.006), while 66.0% lived in a married household (SE = 0.006).

The results of the structural equation model (SEM) are summarized in Table 2, detailing the direct and interaction effects of various predictors on trauma, intracranial volume, tobacco use, cognitive performance, emotional and behavioral problems (CBCL), and reading outcomes.

### 3.1. Parental Employment, Race, and Trauma

Parental employment was found to be significantly associated with lower levels of trauma in children (B = −0.045, SE = 0.011, 95% CI [−0.066, −0.023], p < 0.001). However, consistent with the Minority Diminished Returns (MDRs) framework, the interaction between Black race and parental employment revealed a significant positive association with trauma (B = 0.048, SE = 0.017, 95% CI [0.014, 0.081], p = 0.005), indicating that the protective effect of parental employment was weaker for Black children compared to White children. This suggests that employed Black parents’ children experience higher levels of trauma than their White counterparts, despite similar employment status.

### 3.2. Sociodemographic Predictors of Trauma

Other sociodemographic variables such as age (B = −0.002, SE = 0.009, 95% CI [−0.020, 0.017], p = 0.864), male gender (B = 0.001, SE = 0.009, 95% CI [−0.018, 0.019], p = 0.953), and parental education (B = 0.001, SE = 0.010, 95% CI [−0.020, 0.021], p = 0.936) were not significant predictors of trauma. However, living in a married household was associated with lower trauma levels (B = −0.115, SE = 0.011, 95% CI [−0.135, −0.094], p < 0.001).

### 3.3. Trauma and Intracranial Volume

Trauma was negatively associated with intracranial volume (B = −0.033, SE = 0.009, 95% CI [−0.052, −0.015], p < 0.001), indicating that higher levels of trauma were associated with smaller brain volume in children. This finding underscores the potential long-term neurobiological impacts of trauma on brain development.

### 3.4. Trauma and Tobacco Use

Trauma was positively associated with future tobacco use (B = 0.042, SE = 0.009, 95% CI [0.024, 0.060], p < 0.001), suggesting that children with higher trauma levels were more likely to engage in tobacco use in the future. This relationship highlights the behavioral risks associated with childhood trauma.

### 3.5. Trauma and Emotional/Behavioral Problems

Trauma was also significantly associated with higher CBCL scores, reflecting increased emotional and behavioral problems (B = 0.188, SE = 0.009, 95% CI [0.170, 0.206], p < 0.001). This supports the idea that trauma is a key contributor to behavioral difficulties in children, including symptoms such as anxiety, depression, and aggression.

### 3.6. Trauma and Cognitive Outcomes

Regarding cognitive outcomes, trauma was negatively associated with both total cognitive scores (B = −0.058, SE = 0.009, 95% CI [−0.076, −0.039], p < 0.001) and reading scores (B = −0.046, SE = 0.009, 95% CI [−0.065, −0.028], p < 0.001). These findings indicate that children experiencing higher trauma levels had lower cognitive performance and reading abilities, emphasizing the detrimental effects of trauma on academic achievement and intellectual development.

**Figure 1.**
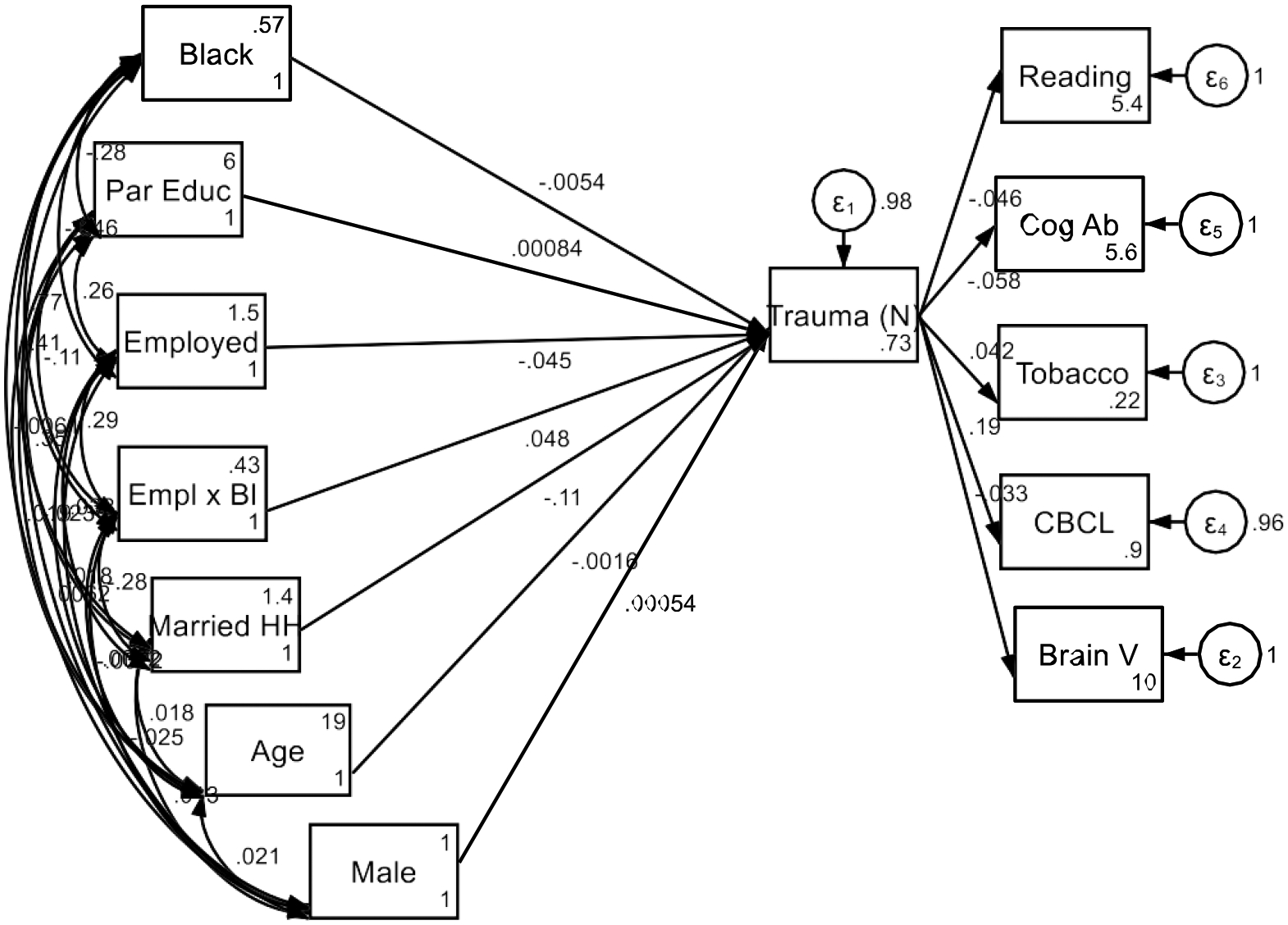
Summary of structural equation models

As shown in Figure 1, the results demonstrate that while parental employment is generally protective against trauma, the benefits are not equally experienced across racial groups. Black children of employed parents continue to face high levels of trauma, which in turn negatively impacts their cognitive, emotional, and behavioral outcomes. These findings provide support for the MDRs framework, showing that socioeconomic resources such as employment do not uniformly translate into reduced adversity across racial groups, especially for Black families.

## 4. Discussion

The primary aim of this study was to explore racial disparities in the protective effects of parental employment on trauma and its subsequent cognitive and behavioral consequences in children. Specifically, we sought to investigate whether the benefits of parental employment—typically associated with lower trauma and better developmental outcomes—were diminished for Black children compared to their White counterparts. This study builds on the Minority Diminished Returns (MDRs) framework, which posits that socioeconomic status (SES) indicators, such as employment, do not yield equal advantages across racial groups. Using the ABCD study’s diverse sample, we assessed the role of trauma as a mediator and examined how racial disparities persist in developmental outcomes despite similar levels of parental employment.

One of the most notable findings of this study is the differential relationship between parental employment and trauma across racial groups. While employment is generally seen as a buffer against adversity, our analysis revealed that Black children with employed parents continued to experience higher levels of trauma and stress compared to their White counterparts. This suggests that the financial security and stability provided by employment does not fully protect Black families from the chronic stressors associated with being racialized in the United States. For many employed Black families, unique stressors—such as workplace discrimination, microaggressions, and the challenges of navigating predominantly White professional environments—can exacerbate trauma experienced by both parents and children. This finding supports the MDRs hypothesis that socioeconomic resources do not equally translate into reduced adversity for Black families due to the additional burdens of racism and discrimination.

Our second aim explored the relationship between trauma and children’s emotional, behavioral, and cognitive outcomes. We found that while parental employment was linked to lower trauma, trauma itself was associated with improved cognitive outcomes— such as reading, cognitive functioning, and lower levels of depression and tobacco use— for both Black and White children. However, consistent with the MDRs framework, because trauma remains more prevalent in the lives of middle-class Black children, the protective effects of parental employment are weaker for them compared to White children. This helps explain why disparities in emotional, behavioral, and cognitive outcomes persist even among upper- and middle-class Black children.

Similarly, Black parents who are employed may report that their children exhibit higher levels of behavioral or emotional issues, such as depression and tobacco use, compared to White children with employed parents. This aligns with the MDRs theory, suggesting that, despite economic stability, middle-class and employed Black families face heightened psychosocial stressors, which may result in more pronounced behavioral and emotional challenges in their children. The elevated trauma and stress levels in these families likely contribute to these difficulties, further demonstrating the limits of SES in protecting Black children from adverse developmental outcomes.

Racism—both structural and interpersonal—emerges as a central factor in explaining the diminished returns of parental employment for Black families. Structural racism in housing, education, employment, and healthcare limits the full benefits that Black families can derive from SES resources. Discriminatory practices in the workforce often lead to higher stress levels, lower job satisfaction, and economic instability for Black parents, even when they are employed. Additionally, Black professionals frequently face microaggressions and exclusion in predominantly White work environments, which compounds the emotional toll on families. The accumulation of these stressors offsets the psychological and economic benefits of employment, leading to increased trauma for Black children. Furthermore, higher-SES Black families often experience added scrutiny and surveillance in their communities, creating a hostile social environment that undermines their children’s emotional well-being and behavioral health.

While this study provides valuable insights into the differential effects of parental employment on trauma and child outcomes, several limitations must be acknowledged. First, the cross-sectional nature of the ABCD data limits our ability to draw definitive causal conclusions about the relationships between parental employment, trauma, and developmental outcomes. Longitudinal studies are necessary to better understand the temporal dynamics of these associations and to capture the long-term impact of parental employment on child development. Second, while we controlled for key variables, additional unmeasured factors—such as neighborhood conditions, parental stress, and experiences of discrimination—may further explain the diminished returns for Black families. Lastly, this study focused on Black and White families, and future research should expand the MDRs framework to explore how parental employment affects other racial and ethnic groups, particularly Latinx, Indigenous, and Asian populations.

The implications of this study are significant for policymakers, educators, and public health practitioners. The findings underscore the need for interventions that address the unique stressors faced by Black families, even when they achieve high levels of SES. Social policies aimed at reducing racial inequalities must go beyond simply increasing access to employment and focus on dismantling broader societal stressors—such as racism, discrimination, and exclusion—that disproportionately affect Black families. Anti-racism initiatives in the workplace, efforts to ensure equity in education and healthcare, and trauma-informed mental health support tailored to Black families are critical steps in addressing these disparities.

## 5. Conclusion

This study highlights the complex relationship between parental employment, trauma, and child developmental outcomes through the lens of racial disparities. While parental employment is often protective against adversity, the diminished returns experienced by Black families reflect the pervasive impact of racism and structural inequalities. Trauma serves as a critical pathway through which these diminished returns manifest, leading to weaker protective effects of parental employment on cognitive, emotional, and behavioral outcomes for Black children. To reduce these disparities, policymakers and practitioners must address the intersection of SES and race, focusing on policies that reduce the systemic stressors faced by Black families. Future research should continue to explore the specific mechanisms driving these diminished returns and evaluate targeted interventions designed to support the well-being of racially marginalized children and their families. Understanding these dynamics is essential for developing effective strategies to combat persistent racial inequalities in child development.

## Figures and Tables

**Table 1.** Descriptive Data Overall

	Mean	SE	95% conf.	interval
				
Age (Month)	9.480	0.005	9.470	9.490
				
	%	SE	95% conf.	interval
Race				
White	0.729	0.006	0.717	0.740
Black	0.271	0.006	0.260	0.283
Gender				
Female	0.476	0.007	0.463	0.489
Male	0.524	0.007	0.511	0.537
Marital Status of the Household				
Unwed Household	0.340	0.006	0.327	0.352
Married Household	0.660	0.006	0.648	0.673

**Table 2.** Summary of Structural Equation Model

Predictor		Outcome	B	SE	95%	CI	P
Age	→	Trauma (N)	−0.002	0.009	−0.020	0.017	0.864
Male	→	Trauma (N)	0.001	0.009	−0.018	0.019	0.953
Parental Education (Years)	→	Trauma (N)	0.001	0.010	−0.020	0.021	0.936
Married Household	→	Trauma (N)	−0.115	0.011	−0.135	−0.094	< 0.001
Black	→	Trauma (N)	−0.005	0.017	−0.039	0.028	0.756
Parental Employed	→	Trauma (N)	−0.045	0.011	−0.066	−0.023	< 0.001
Black × Parental Employment	→	Trauma (N)	0.048	0.017	0.014	0.081	0.005
Trauma (N)	→	Intracranial Volume	−0.033	0.009	−0.052	−0.015	< 0.001
Trauma (N)	→	Tobacco Use (Future)	0.042	0.009	0.024	0.060	< 0.001
Trauma (N)	→	CBCL Score	0.188	0.009	0.170	0.206	< 0.001
Trauma (N)	→	Total Com (Cognitive, Age Corrected)	−0.058	0.009	−0.076	−0.039	< 0.001
Trauma (N)	→	Reading (Age Corrected)	−0.046	0.009	−0.065	−0.028	< 0.001
